# Behavioral, Sociocultural, and Institutional Barriers to Dengue Prevention and Control Among Rural Communities in the Peruvian Amazon

**DOI:** 10.3390/healthcare14121715

**Published:** 2026-06-15

**Authors:** Miguel A. Arce-Huamani, Williams Carrascal-Astola, Brissa C. Haro-Vásquez, Brishel Navarro-Ochoa, Karin M. Chuquihuara-Guerrero, Amir M. Pineda-Chuquiyauri, Lesly C. Paucar-Sanchez, Maritza M. Ortiz-Arica

**Affiliations:** Public Health, Surveillance and Applied Research Group, Academic Program of Human Medicine, Faculty of Health Sciences, Universidad Privada Norbert Wiener, Lima 15046, Peru; williams.carrasca@uwiener.edu.pe (W.C.-A.); brihv2917@gmail.com (B.C.H.-V.); brishel02@gmail.com (B.N.-O.); karinchg09@gmail.com (K.M.C.-G.); pinedachuquiyauriamir@gmail.com (A.M.P.-C.); les.pau.2004@gmail.com (L.C.P.-S.); ma.ortiz.arica@gmail.com (M.M.O.-A.)

**Keywords:** dengue, *Aedes aegypti*, vector control, rural health, Amazonian communities

## Abstract

**Background/Objectives**: Dengue prevention in rural Amazonian communities is shaped by knowledge, household feasibility, sociocultural dynamics, institutional continuity, and trusted communication. This study explored behavioral, sociocultural, and institutional barriers to dengue prevention and control in rural communities of the Peruvian Amazon. **Methods**: An exploratory qualitative study with an ethnographic orientation, informed by the Communication for Behavioural Impact (COMBI) framework, was conducted in three anonymized rural settlements in San Martín, Peru. The qualitative corpus included 120 adults, 84 in-depth interviews, six focus group discussions with 36 participants, 22 household and community observation records, 13 institutional communication materials, and seven local operational documents. Data were analyzed using an inductive thematic approach and triangulated across participant profiles, settlements, and sources. **Results**: Dengue was widely recognized as a mosquito-borne disease, but the central finding was a gap between general awareness and practical, routine application. Participants’ understanding of breeding sites, warning signs, and feasible source reduction was uneven. Prevention was mainly reactive, increasing after nearby cases, alerts, or fumigation, but weakening when risk was not visible. Irregular water supply, water storage, waste accumulation, gendered domestic labor, competing household priorities, reluctance to confront neighbors, and intermittent institutional action limited sustained prevention. Fumigation was perceived as the most visible institutional response, while communication was more credible when mediated by trusted local actors. **Conclusions**: Dengue prevention requires locally feasible household practices, safe water-storage guidance, trusted communicators, neighborhood coordination, continuous pre-outbreak engagement, and intersectoral support.

## 1. Introduction

Dengue is no longer a geographically contained public health problem in Peru. In 2023, the country reported 251,605 confirmed cases, the highest incidence recorded nationally, and 60 districts detected local community transmission for the first time [1]. In the northern Peruvian Amazon, recent entomological surveillance identified *Aedes aegypti* populations in 29 of 30 surveyed sites across a rural–urban gradient, indicating that rural communities historically considered peripheral to dengue transmission are now exposed to sustained vector-borne disease risk [2]. Loreto, an Amazonian department in northeastern Peru that includes Iquitos and extensive riverine settlements, illustrates the operational difficulty of dengue control in remote settings. In this region, routine vector control remains dependent on public-sector actions whose costs have been described as high and difficult to sustain because the effect of current methods is short-lived [3]. Earlier qualitative work in Iquitos, Loreto, showed that caregivers recognized dengue symptoms and larval-control practices, but misconceptions about mosquito behavior, perceived powerlessness, and the belief that health services or government were mainly responsible weakened preventive action [4]. Taken together, these Peruvian findings show that dengue control in Amazonian settings is simultaneously ecological, behavioral, and institutional.

Peruvian evidence from Villa El Salvador, a large urban district in Lima, has quantified dengue-related knowledge, attitudes, and practices among adults, showing the value of KAP approaches while also underscoring that knowledge alone does not explain how prevention is sustained in everyday household life [5]. However, rural Amazonian communities differ from urban coastal settings because dengue prevention is shaped by intermittent water supply, domestic water storage, transport limitations, dispersed households, and irregular institutional presence. These conditions may make standard prevention messages difficult to apply, even when communities recognize dengue as a relevant health threat.

Evidence from neighboring South American settings also suggests that dengue prevention depends on more than individual knowledge. Household research in Ecuador found that communities used multiple mosquito-control strategies, but prevention decisions were strongly shaped by cost, perceived effectiveness, and economic limitations [6]. In Colombia, stakeholders involved in vector control emphasized disjointed institutional efforts, insufficient resources, and sociocultural and political constraints as obstacles to effective surveillance and response [7]. These studies suggest that dengue prevention fails not only because of limited information, but because household practices, local meanings, institutional trust, and service capacity interact unevenly.

Despite this regional evidence, there remains limited qualitative research explaining how rural Amazonian households understand, negotiate, and sustain dengue prevention in daily life. This gap is important because dengue risk is expanding into territories where ecological vulnerability, domestic water practices, institutional reach, and community experience may not align with standard control messages. A locally grounded qualitative approach is therefore necessary to clarify why preventive recommendations are accepted, adapted, delayed, or abandoned in real community contexts. This study aimed to explore behavioral, sociocultural, and institutional barriers to dengue prevention and control among rural communities in the Peruvian Amazon.

## 2. Materials and Methods

### 2.1. Study Design and Setting

We conducted an exploratory qualitative study with an ethnographic orientation, informed by the Communication for Behavioural Impact (COMBI) framework. The study was conducted over a one-month period in 2025 in three anonymized rural Amazonian settlements in the department of San Martín, Peru. This design was selected to understand how behavioral, sociocultural, institutional, and communication-related barriers shape dengue prevention and control in everyday community life.

The settlements were purposively selected to capture contrasting rural Amazonian contexts relevant to dengue prevention. Selection was based on four criteria: (1) rural Amazonian location with perceived dengue risk or previous dengue-prevention activities; (2) variation in water availability, transport access, and proximity to health services; (3) feasibility and safety for qualitative fieldwork; and (4) permission from local leaders or community representatives. The three settlements were selected because they represented distinct rural Amazonian conditions: roadside agricultural access, riverine dispersion with irregular water access, and more remote rural isolation with limited transport to health services. These settlements were not selected to be statistically representative of all rural communities in San Martín. Rather, they were selected to provide analytical variation in the household, community, and institutional conditions that may influence dengue prevention.

To improve transferability while preserving confidentiality, we provide additional contextual information using approximate ranges rather than identifiable settlement names. Settlement A was a roadside agricultural community of approximately 75–90 households, with an estimated population of 320–380 inhabitants, located around 2 h from the nearest health facility. This community had relatively greater road access and commercial movement, although dengue-prevention activities were still affected by domestic workload, stored water, and waste accumulation. Settlement B was a dispersed riverine community of approximately 85–100 households, with an estimated population of 390–450 inhabitants, located around 2.5 h from the nearest health facility. In this settlement, household water storage was common because water access was irregular and water was needed for cooking, washing, and other domestic uses. Settlement C was a more remote rural community of approximately 95–110 households, with an estimated population of 450–500 inhabitants, located around 3 h from the nearest health facility. Transport was less frequent and more costly, and access to health services was more difficult during rainfall, night-time symptoms, or periods of poor road conditions. These contextual details are intentionally approximate to protect community confidentiality while allowing readers to assess the transferability of the findings to other rural Amazonian settings.

The settlements were anonymized to protect confidentiality and to avoid the identification of small rural communities.

### 2.2. Participants, Recruitment, and Sampling

The study included adult participants aged 18 years or older who lived permanently in the selected rural communities or who had a direct community, educational, health-service, or local institutional role related to dengue prevention and control. Participants included household adults and primary caregivers, community leaders, neighborhood representatives, local authorities, community health agents, health promoters, health personnel, vector-control workers, teachers, school representatives, youth or community organizers, and municipal or other local institutional actors.

A purposive and maximum-variation sampling strategy was used to capture diverse perspectives across household, community, and institutional domains. Recruitment sought variation by settlement, community role, proximity to health services, water-storage conditions, and experience with dengue-related prevention activities. Potential participants were identified through community entry points, local leaders, health promoters, and snowball referrals when additional perspectives were needed. Participation was voluntary, and refusal to participate did not affect access to any service or community activity.

The final qualitative corpus comprised 120 adult participants: 49 from Settlement A, 38 from Settlement B, and 33 from Settlement C. The participant profile included 71 household adults or primary caregivers, 15 community leaders or local authorities, 11 community health agents or health promoters, 10 health personnel or vector-control workers, 7 teachers or community organizers, and 6 municipal or local institutional actors.

Because this was a qualitative study, no statistical sample-size calculation was performed. Sampling continued until thematic sufficiency was reached. This was assessed progressively during fieldwork through review of field notes, preliminary coding summaries, and a coding matrix organized by settlement, participant role, and the four analytical domains: behavioral, sociocultural, institutional, and communication/community response. The research team considered thematic sufficiency to have been reached when additional interviews, focus group discussions, and observations no longer added substantially new information to the main categories, and when the final materials mainly confirmed or nuanced previously identified patterns. The last stage of data collection was used to check consistency across settlements and to identify contrasting or negative cases.

### 2.3. Research Team, Reflexivity, and Data Collection

Data were collected by trained members of the research team affiliated with Universidad Privada Norbert Wiener, Peru. The team had experience in public health, community-based research, and dengue-prevention topics. Before fieldwork, the research team reviewed the study objectives, standardized the interview and focus group guide, discussed culturally appropriate wording, and agreed on procedures for informed consent, probing, note-taking, and protection of confidentiality.

The researchers introduced themselves as academic investigators and not as personnel responsible for local vector-control decisions or municipal enforcement. No interviewer had a direct clinical, administrative, or supervisory relationship with the participants. Potential researcher-related bias was addressed by emphasizing the voluntary nature of participation, explaining that there were no right or wrong answers, encouraging participants to describe both positive and negative experiences, and discussing field impressions reflexively within the research team during data collection and analysis.

Data were collected through individual in-depth interviews, focus group discussions, household and community observations, and review of institutional communication materials and local operational documents. The final corpus included 84 individual in-depth interviews, 6 focus group discussions involving 36 participants, 22 household and community observation records, 13 institutional communication materials, and 7 local plans, meeting minutes, or operational documents.

Individual interviews lasted approximately 20–30 min, while focus group discussions lasted approximately 60 min. Interviews and focus group discussions were conducted in private or comfortable community settings selected to protect confidentiality and reduce interruptions. With prior informed consent, sessions were audio-recorded and complemented with field notes. Questions were open-ended and adapted to the participant’s age, educational level, role, and local context.

The semi-structured qualitative guide explored four domains: behavioral factors, sociocultural factors, institutional barriers, and communication/community response. The behavioral domain explored knowledge, attitudes, perceived risk, warning signs, and household preventive practices related to dengue. The sociocultural domain examined beliefs, customs, household organization, gendered prevention roles, community priorities, and interpersonal dynamics related to neighborhood-level control. The institutional domain explored access to health services, perceived state presence, fumigation, health campaigns, local coordination, and barriers to surveillance or control. The communication domain examined channels, clarity, trust, local adaptation, and the degree of alignment or mismatch between institutional messages and feasible household action.

Observations focused on domestic and peri-domestic spaces, water-storage practices, potential mosquito breeding sites, waste accumulation, community interaction, and visible traces of prevention activities. Communication materials, posters, local messages, meeting records, and operational documents were reviewed to compare institutional recommendations with community interpretation and reported practices.

### 2.4. COMBI-Informed Analytical Framework

The COMBI framework was used as a formative planning lens rather than as an intervention model or evaluation framework. No COMBI intervention was implemented or assessed in this study. Instead, COMBI informed the organization and interpretation of findings by helping the research team identify barriers that could affect future communication, community mobilization, and behavior-change strategies for dengue prevention.

Findings were mapped to five COMBI-informed planning domains. First, behavioral objectives included specific and observable household actions, such as identifying breeding sites, washing or covering water containers, eliminating discarded objects, and seeking timely care for warning signs. Second, message and channel strategy included clarity of messages, recall of prevention recommendations, use of radio, loudspeakers, WhatsApp, schools, posters, and household visits, and the credibility of each channel. Third, community mobilization included the role of community leaders, teachers, health promoters, neighbors, and collective campaigns. Fourth, administrative and institutional mobilization included health-sector presence, fumigation, municipal coordination, vector-control activities, and continuity of prevention before outbreaks. Fifth, enabling contextual conditions included water-storage needs, waste management, transport barriers, household workload, and other structural constraints that shaped whether recommended behaviors were feasible.

This mapping allowed the analysis to move beyond a descriptive list of barriers and to identify which barriers should be addressed by future COMBI-informed dengue-prevention interventions.

### 2.5. Data Analysis and Trustworthiness

Data were analyzed using an inductive thematic approach. Audio recordings were transcribed verbatim, and transcripts, field notes, observation records, communication materials, and local documents were organized as a single qualitative corpus. ATLAS.ti 25 (ATLAS.ti Scientific Software Development GmbH, Berlin, Germany, 2025) was used to organize the corpus, support coding, compare fragments across participant profiles and settlements, and construct thematic relationships.

The analysis began with repeated reading of the material to identify initial meaning units related to dengue knowledge, perceived risk, household feasibility, sociocultural priorities, institutional presence, and communication processes. Initial coding was conducted independently by two researchers on a subset of transcripts and field materials. The researchers then compared codes, discussed discrepancies, refined definitions, and developed a shared coding matrix. After agreement on the coding structure, the remaining material was coded using the refined framework, while allowing new codes to emerge when needed.

Coding matrices were used to compare themes across settlements, participant roles, and data sources. Interview and focus group accounts were compared with observation records and reviewed institutional materials to identify convergences, contradictions, and contextual explanations. Thematic sufficiency was assessed through this matrix-based comparison, with attention to whether new materials added new themes or mainly reinforced existing analytical categories.

Methodological rigor was strengthened through triangulation across interviews, focus group discussions, observations, communication materials, and operational documents; reflexive discussion among researchers; maintenance of field notes; systematic documentation of coding decisions; and use of anonymized illustrative excerpts. Credibility was supported by comparing community, household, and institutional perspectives. Transferability was supported through detailed contextual description of the study setting, participants, and fieldwork procedures. Confirmability was supported by preserving an audit trail of transcripts, codes, field notes, coding matrices, and analytical decisions.

### 2.6. Ethical Considerations

The study protocol was approved by the Institutional Committee for Ethics and Scientific Integrity of Universidad Privada Norbert Wiener, Lima, Peru (CIEIC-UPNW; approval No. A0040-2025; approval date: 22 May 2025). All participants received clear information about the study objectives, procedures, voluntary nature of participation, approximate duration, potential risks and use of the information provided. Written informed consent was obtained before data collection.

Participation was voluntary, and participants could decline to answer any question or withdraw from the study at any time without consequences. The study involved minimal risk because no invasive procedures or clinical interventions were performed. However, because some interviews could address sensitive experiences related to illness, institutional response or community conflict, interviews were conducted respectfully and participants’ autonomy was prioritized throughout the fieldwork.

Confidentiality was strictly protected. Names, personal identifiers and community identifiers were removed from transcripts and reports. Settlements were anonymized as Settlement A, Settlement B and Settlement C. Audio recordings, transcripts, field notes and documentary materials were stored securely and were accessible only to the research team. Findings are reported in aggregate thematic form, using anonymized descriptions to avoid identification of individual participants or small rural communities.

### 2.7. Data Availability and Use of Generative Artificial Intelligence

No generative artificial intelligence tools were used to generate, modify or fabricate field data, participant accounts, transcripts, codes, quotations or analytical findings. Generative artificial intelligence was used only for language refinement, formatting and editorial improvement of the manuscript. Final responsibility for the scientific content, interpretation and integrity of the manuscript rests entirely with the authors.

## 3. Results

The analysis generated six interconnected themes that explain why dengue prevention was difficult to sustain in everyday community life: (1) dengue knowledge was present but not fully actionable; (2) prevention was reactive and constrained by household feasibility; (3) sociocultural priorities shaped participation; (4) community coexistence limited neighborhood-level control; (5) institutional action and communication were perceived as intermittent; and (6) trusted local mediation was essential for translating messages into feasible household action. These themes were derived from triangulation across interviews, focus group discussions, household and community observations, institutional communication materials, and local operational documents. Participant quotations are presented anonymously and were translated from Spanish into English. Quotations were lightly edited for clarity without altering their substantive meaning. Table 1 presents the thematic synthesis of the main barriers to dengue prevention and control.

### 3.1. Dengue Knowledge Was Present but Not Fully Actionable

Across the corpus, dengue was widely recognized as a mosquito-borne disease, and participants commonly associated it with fever, headache, body pain, and general weakness. This basic awareness was shared across settlements and participant profiles. However, knowledge became less consistent when participants described where mosquitoes breed, how risk accumulates inside households, or which warning signs should prompt urgent consultation. The main finding was therefore not a complete absence of knowledge, but a gap between general awareness and practical, routine application.

One participant summarized this distinction between knowing about dengue and recognizing breeding sites in everyday household spaces: “We know that dengue is carried by the mosquito; that much we know. But sometimes we do not realize where it is breeding, because it can be in a small bottle cap, on a leaf, or in anything that collects water” (Household participant).

Participants from Settlement A, where commercial movement and institutional contact were more frequent, tended to recall campaign messages more easily. In Settlement B, accounts more often emphasized the difficulty of preventing mosquitoes when water had to be stored for daily use. In Settlement C, distance from formal health services made symptom interpretation and care-seeking decisions more central to household narratives. These contrasts suggest that the same prevention message acquired different meanings depending on water availability, transport, institutional proximity, and previous experience with dengue.

A recurrent tension emerged between what households knew they “should” do and what they considered feasible. Many participants recalled recommendations such as washing containers or eliminating breeding sites, yet observation records identified buckets, animal water recipients, plant containers, discarded objects, and shaded patios where water could accumulate. This contrast between reported knowledge and observed domestic conditions suggested that prevention was often understood as a general duty, but was not always translated into detailed inspection of small, ordinary household spaces.

### 3.2. Prevention Was Reactive and Constrained by Household Feasibility

Prevention was commonly activated by visible risk. Concern increased after nearby cases, school alerts, rumors of severe illness, fumigation visits, or health-brigade activities. During those periods, households were more likely to clean patios, remove containers, wash water recipients, or participate in community campaigns. In periods without visible cases, preventive routines became less consistent.

As one participant explained: “When we hear that someone nearby has dengue, only then does everyone hurry to clean, throw away stagnant water, and cover the buckets. But when there are no cases, people become confident and let it pass” (Household participant).

This pattern suggests that dengue prevention was largely event-driven. Dengue was perceived as serious, particularly for children, older adults, and people with previous illness; however, the sense of urgency depended on whether risk was socially visible. When cases occurred nearby or when fumigation teams arrived, dengue became more present in community conversations. When no cases were visible, prevention competed with daily work, domestic responsibilities, agricultural activities, childcare, food needs, and other household priorities.

Water storage was one of the most important constraints shaping preventive practices. In households affected by irregular supply, storing water was necessary for cooking, washing, animals, and other domestic needs. In this context, recommendations to eliminate containers or avoid stored water were sometimes perceived as unrealistic. Water storage was not merely a risky behavior; it was a household adaptation to service limitations.

One participant described this tension clearly: “Here we have to store water because it does not always come. It is not that we want to keep buckets full, but that water is used for cooking, washing, and for the house. That is why we cannot just throw it away” (Household participant).

### 3.3. Sociocultural Priorities Shaped Participation

Dengue prevention was embedded in broader household and community responsibilities. Participants did not necessarily reject prevention messages; rather, prevention competed with work, childcare, food preparation, water collection, transport, school responsibilities, and other immediate needs. In several accounts, dengue prevention was described as something important but difficult to maintain when no case was visible and when daily subsistence tasks were more urgent.

A participant expressed this competition between prevention and daily responsibilities as follows: “We would like to keep everything clean all the time, but we also have to go to work, cook, look after the children, bring water, and do the housework. Sometimes the day is not enough, and cleaning is left for later” (Household participant).

Prevention work was also strongly gendered. Women, mothers, grandmothers, and primary caregivers were frequently described as those responsible for washing containers, cleaning patios, managing stored water, and monitoring children’s symptoms. This made prevention dependent on repetitive and often invisible domestic labor. The finding suggests that dengue-prevention strategies may unintentionally reinforce gendered responsibilities if they address “the household” without recognizing who actually performs the daily prevention work.

### 3.4. Community Coexistence Limited Neighborhood-Level Control

Participants recognized that dengue risk extended beyond individual households. They frequently mentioned neighboring patios, uncovered containers, waste accumulation, abandoned objects, and shared spaces as possible sources of mosquitoes. However, they were often reluctant to directly confront neighbors or report specific households because doing so could create interpersonal conflict.

One participant described this reluctance in practical terms: “Sometimes you see that a neighbor has things lying around or buckets with water, but you cannot say much, because some people get upset or think that you are interfering in their life” (Community participant).

This finding shows that community coexistence had a double role. On one hand, it facilitated collective campaigns when leaders, schools, or health promoters called for participation. On the other hand, it limited everyday correction of risk conditions when action required pointing out a neighbor’s waste, uncovered water, or stagnant containers. In this context, neighborhood-level prevention cannot rely only on individual complaints. It requires non-punitive community mechanisms, such as collective clean-up days, shared inspection routes, and locally legitimate mediation.

### 3.5. Institutional Action and Fumigation Were Perceived as Intermittent

Fumigation was the most visible institutional response and was often interpreted as evidence that authorities were acting. Participants frequently associated official dengue control with fumigation, health campaigns, and visits by health personnel or vector-control teams. However, these actions were often perceived as more frequent during outbreaks, alerts, or periods of visible risk, rather than as continuous prevention.

A participant explained this perception as follows: “When they come to fumigate, we feel that something is finally being done about dengue. But afterward they leave and do not come back, until more sick people appear again” (Community participant).

This finding helps explain why fumigation occupied such a strong symbolic place in community narratives. It was visible, immediate, and easier to recognize than larval-source reduction or household inspection. However, this visibility may also reinforce dependence on external action if fumigation is not accompanied by sustained household education, water-container management, waste control, and community follow-up. The analytical issue is therefore not simply that communities “believe in fumigation,” but that fumigation represents institutional presence in settings where other forms of support are perceived as intermittent.

### 3.6. Trusted Local Mediation Was Essential for Translating Messages into Feasible Action

Communication about dengue reached communities through several channels, including radio, loudspeakers, WhatsApp, schools, health talks, posters, and direct visits. However, no single channel reached all groups equally. Radio and loudspeakers were more useful for some older adults or households with limited internet access. WhatsApp and school-based communication reached other groups, particularly younger adults and families with school-age children. Posters were less influential when they were not explained by trusted local actors.

Messages were more credible when mediated by people with local legitimacy, such as community leaders, teachers, health promoters, and familiar health personnel. Trust depended on proximity, continuity, and perceived commitment. Participants were more likely to consider messages useful when they were translated into concrete household examples, such as how to cover stored water when covers are limited, how to identify small containers, how to manage animal water recipients, and what to do when fever appears at night or during rainfall.

One participant highlighted the importance of trusted local explanation: “When the health promoter or someone we know from the community speaks to us, we understand better, because they speak clearly and know how we live here” (Community participant).

Overall, the findings suggest that communication should move beyond simply repeating standard messages. In these rural Amazonian settings, prevention messages became meaningful when they were explained by trusted local actors and adapted to the material conditions of household life.

A summary of the main themes, qualitative findings, and interpretive implications is provided in Supplementary Table S1.

### 3.7. Integrated Interpretation for Future COMBI-Informed Planning

Overall, dengue prevention and control in these rural Amazonian communities were shaped by the interaction between practical knowledge, event-driven risk perception, household feasibility, gendered prevention roles, intermittent institutional presence, and locally mediated communication. The central challenge was not simply lack of information. Rather, public health recommendations were often difficult to sustain under the everyday conditions of rural Amazonian households.

These findings point to practical implementation priorities: working with existing water-storage practices instead of assuming that stored water can simply be eliminated; redistributing prevention tasks beyond women and primary caregivers; creating neighborhood-level mechanisms that do not depend on direct confrontation between neighbors; strengthening trusted local communication; and maintaining pre-outbreak prevention rather than relying mainly on visible emergency responses. These priorities are consistent with the formative use of behavior-change planning frameworks such as COMBI, but the study did not implement or evaluate a COMBI intervention.

## 4. Discussion

This study shows that dengue prevention and control in rural Amazonian communities cannot be understood only as a matter of individual knowledge or willingness to follow health recommendations. Participants generally recognized dengue as a mosquito-borne disease and were familiar with basic prevention messages; however, this awareness did not consistently translate into sustained household practices. The main issue was not the absence of information, but the difficulty of converting general recommendations into feasible daily actions in contexts marked by intermittent water supply, domestic workload, limited waste management, transport barriers, and episodic institutional presence. Prevention was largely reactive, intensifying after nearby cases, alerts, or fumigation activities, but weakening when risk was no longer socially visible. These findings suggest that dengue control in rural Amazonian settings depends on the interaction between actionable knowledge, household feasibility, sociocultural organization, trust in local actors, and continuity of institutional support.

The gap between dengue awareness and actionable prevention is consistent with previous evidence showing that knowledge alone does not ensure routine source reduction. In rural Colombia, López-Saleme et al. (2025) reported that residents had acceptable knowledge of dengue-control practices, but knowledge and attitudes regarding vector characteristics remained limited and sources of risk in the immediate environment were still neglected [8]. Similarly, Jaramillo-Ramirez et al. (2025) found that people in an endemic Colombian municipality had general knowledge of arboviral infections but lacked more specific understanding of transmission and self-protection; importantly, knowledge of water-tank cleaning was associated with lower odds of mosquito presence in the household [9]. These findings support our interpretation that general awareness must be translated into concrete, repeated, and locally feasible household practices.

Prevention was also shaped by visible risk. Household cleaning, patio organization, and container management increased after nearby cases, campaigns, or fumigation, but weakened when cases were not apparent. This pattern is consistent with qualitative evidence from Nepal, where perceived severity and susceptibility during recurrent outbreaks motivated engagement, while delayed action and inconsistent source reduction remained important barriers [10]. In Colombia’s Orinoquia region, stakeholders similarly criticized vector-control responses that tended to wait for outbreaks before intervening [7]. Our findings therefore suggest that dengue prevention should not depend only on outbreak-triggered mobilization. Rural prevention strategies need routine pre-outbreak practices that remain meaningful even when dengue is not socially visible.

Material constraints were central to prevention feasibility. Water storage, containers, patios, and waste were not simply behavioral problems; they reflected everyday household adaptations to irregular services and limited infrastructure. This interpretation is supported by Ghimire and Singh (2025), who identified inadequate water supply as a barrier to dengue management because households stored water in ways that could create breeding sites [10]. It is also consistent with rural Colombian evidence showing that waste-management problems, limited education, and mosquitoes in water tanks were perceived as relevant barriers despite acceptable knowledge of control practices In rural Amazonian communities, recommendations to eliminate containers or avoid stored water may be unrealistic unless they are accompanied by safe covers, regular waste collection, feasible container-management guidance, and structural improvements in water access.

The findings also show that dengue prevention depends on gendered domestic labor. Women, mothers, grandmothers, and primary caregivers were repeatedly associated with washing containers, cleaning patios, managing stored water, and monitoring children’s symptoms. This aligns with the qualitative meta-synthesis by Mungall-Baldwin et al. (2022), which concluded that women are a key human resource for household and community-based dengue prevention, although their participation has often not been gender-equitable, gender-sensitive, or transformative [11]. Our findings extend this evidence by showing how routine prevention tasks become part of repetitive and low-visibility domestic work. If prevention responsibilities are not redistributed across households and communities, dengue-control strategies may unintentionally increase the burden on women and caregivers.

Community coexistence both enabled and limited dengue prevention. Participants recognized that breeding sites could persist in neighboring patios, shared spaces, uncovered containers, or accumulated waste, but they often avoided direct complaints to prevent interpersonal conflict. This finding is consistent with evidence that community engagement in dengue control depends on stakeholder participation, partner communication, and continuity across implementation stages [12]. It also helps explain why individual household action is insufficient when risk conditions are distributed across neighboring or shared environments. Public health programs should therefore create non-punitive mechanisms for collective inspection, neighborhood clean-up, and community mediation, so that correcting risk conditions does not depend on direct confrontation between neighbors.

Institutional action was perceived as intermittent, and fumigation occupied a strong symbolic place in community accounts. This does not mean that communities misunderstood dengue control. Rather, fumigation was visible, immediate, and interpreted as evidence that authorities were acting. Similar patterns have been reported in Colombia, where stakeholders described ultra-low-volume spraying as part of Aedes control but also emphasized disjointed institutional efforts, insufficient resources, and weak community engagement.

Institutional action was perceived as intermittent, and fumigation occupied a strong symbolic place in community accounts. This finding agrees with Jaramillo-Ramirez et al. (2025), who reported that participants in Colombia favored interventions by the Health Secretariat, especially insecticide spraying [9]. Jaramillo-Ramirez et al. (2024) also found that Aedes control in Colombia included ultra-low-volume spraying in public spaces, while stakeholders emphasized disjointed institutional efforts, insufficient resources, and weak community engagement [7]. When fumigation is not accompanied by larval-source reduction, water-container management, practical household education, and follow-up, it may reinforce dependence on external outbreak-period responses. For rural Amazonian communities, institutional legitimacy should therefore be built through continuous prevention, not only through visible emergency action.

Health communication reached households through radio, loudspeakers, WhatsApp, schools, posters, health talks, and direct visits, but messages were more credible when explained by trusted local actors [13]. This finding is consistent with Samsudin et al. (2024), who emphasized that effective dengue communication should include clear messages and incorporate residents’ perspectives [14]. Home-to-home visits and awareness programs were also considered useful for public engagement in Nepal [10]. In our study, the key issue was not simply which channel was used, but whether messages were translated into practical examples that made sense for rural households, such as how to manage stored water, small containers, animal water recipients, and night-time fever decisions.

Barriers to health-care access during suspected dengue symptoms limited timely consultation despite awareness of warning signs. Ng et al. (2023) found that health-seeking decisions among dengue patients were influenced not only by knowledge, but also by perceived symptom severity and social circumstances such as childcare [15]. Rajapaksha et al. (2023) similarly reported that adequate health-seeking and dengue-prevention behaviors were not universal in a highly endemic setting [16]. These findings support our interpretation that care-seeking in remote rural Amazonian communities is not only a cognitive decision, but also a territorial and logistical process. Dengue education should therefore be paired with realistic access pathways, including guidance for night-time fever, referral options, transport coordination, and clear thresholds for urgent consultation.

From an implementation perspective, the findings point to practical priorities that stand independently of any single framework: work with existing water-storage practices instead of treating stored water only as non-compliance; redistribute prevention tasks beyond women and primary caregivers; build neighborhood mechanisms that avoid direct confrontation; strengthen trusted local communication; and maintain prevention before outbreaks occur [17]. These priorities are compatible with formative behavior-change planning, including COMBI-informed approaches, but this study did not implement or evaluate a COMBI intervention. Therefore, the findings should be understood as formative qualitative evidence for designing future community-based dengue-prevention strategies, not as evidence of intervention effectiveness.

This study contributes to the literature by showing that dengue prevention in rural Amazonian communities is not limited by lack of awareness alone. Rather, prevention is shaped by the interaction between actionable knowledge, household feasibility, gendered domestic labor, community coexistence, institutional continuity, and locally mediated trust. Although qualitative findings are not statistically generalizable, they offer analytical transferability to other rural, peri-urban, or Amazonian settings where dengue transmission intersects with intermittent water supply, limited transport, dispersed households, weak waste management, and episodic institutional presence.

This study has several strengths. First, it used a broad and diverse qualitative corpus that included household adults, caregivers, community leaders, health promoters, health personnel, teachers, and local institutional actors from three rural Amazonian settlements with different territorial and service-access characteristics. Second, the analysis was strengthened through triangulation across in-depth interviews, focus group discussions, household and community observations, communication materials, and local operational documents. Third, the findings provide practical implementation targets for future dengue-prevention strategies. However, some limitations should be acknowledged. Because this was a qualitative study, the findings should not be interpreted as prevalence estimates, measures of association, or evidence of causal relationships. Social desirability and recall bias may have influenced participants’ accounts of prevention practices and institutional experiences. Participants may have over-reported socially expected actions, such as cleaning patios or covering containers, and under-reported periods of inaction, especially because dengue prevention is publicly valued and fumigation is interpreted as an important institutional response. In addition, settlement anonymization, necessary to protect confidentiality in small rural communities, limited the amount of contextual detail that could be reported. Finally, the study did not evaluate an intervention, entomological outcomes, or longitudinal changes in dengue transmission; therefore, the findings should be understood as formative evidence for future public health strategies rather than proof of intervention effectiveness.

## 5. Conclusions

This qualitative study showed that dengue prevention and control in rural Amazonian communities of San Martín, Peru, are shaped by a complex interaction of behavioral, sociocultural and institutional barriers. Participants generally recognized dengue as a mosquito-borne disease and were familiar with basic prevention messages; however, this knowledge was not always translated into sustained household practices. Prevention tended to intensify when risk became visible through nearby cases, campaigns or fumigation, but weakened during periods without apparent transmission. Material constraints, particularly irregular water supply, water storage, containers, patios and waste accumulation, limited the feasibility of routine source reduction. In this context, stored water should not be interpreted only as an individual risk behavior, but also as a domestic response to structural service limitations.

The study also found that dengue prevention was embedded in gendered household labor, community coexistence norms and intermittent institutional presence. Women, mothers, grandmothers and primary caregivers carried much of the routine prevention work, while neighborhood-level control was constrained by the reluctance to confront others about breeding sites or waste accumulation. Fumigation was perceived as the most visible form of institutional response, but this visibility may reinforce dependence on external action if it is not accompanied by continuous larval-source reduction, practical education and household follow-up. Health communication reached communities through radio, loudspeakers, WhatsApp, schools, posters and direct visits, but messages were more credible and useful when mediated by trusted local actors and adapted to concrete domestic situations.

These findings support the need for future COMBI-informed dengue strategies that define specific, observable and locally feasible behavioral objectives. Such strategies should prioritize safe water-storage practices, practical household demonstrations, locally trusted communicators, neighborhood-level coordination, pre-outbreak community engagement and intersectoral support for water and waste-management constraints. However, the present findings should not be interpreted as evidence of the effectiveness of a COMBI intervention, because no intervention was implemented or evaluated. Rather, this study provides formative qualitative evidence to guide the design of future dengue-prevention policies and community-based interventions in rural Amazonian and similar resource-constrained settings.

## Figures and Tables

**Table 1:** Thematic synthesis of barriers to dengue prevention and control.

Main Theme	Qualitative Finding	Interpretation
**Knowledge was present but not fully actionable**	Participants recognized dengue as mosquito-borne, but practical understanding of breeding sites, warning signs and feasible source reduction was uneven.	The main limitation was not lack of awareness, but difficulty translating general messages into routine domestic practices.
**Prevention was reactive and constrained by feasibility**	Cleaning and concern increased after nearby cases, alerts or fumigation, but routine prevention weakened during periods without visible risk. Irregular water supply made water storage necessary.	Preventive action was shaped by visible risk and by material conditions that limited what households could realistically sustain.
**Sociocultural priorities shaped participation**	Prevention competed with work, childcare, food needs, water collection, transport and school responsibilities. Women and caregivers carried most routine prevention tasks.	Dengue prevention was one priority among many and was often embedded in gendered domestic labor.
**Community coexistence limited neighborhood-level control**	Participants recognized risk in neighboring spaces but avoided direct complaints about waste, stagnant water or uncovered containers because of possible conflict.	Social cohesion facilitated collective campaigns but discouraged individual reporting.
**Institutional action and communication were perceived as intermittent**	Fumigation, health talks and visits were more visible during outbreaks or alerts. Messages were remembered but not always adapted to local constraints.	Institutional response was experienced as reactive, and communication achieved recall more often than sustained practice.

Note: Themes are presented as interpretive qualitative findings derived from triangulation across interviews, focus group discussions, observations and reviewed materials. They should not be interpreted as frequencies, prevalence estimates, statistical rankings, measures of relative importance or causal associations. Bold text indicates the main themes generated from the thematic analysis.

## Data Availability

Because this was a qualitative study involving potentially identifiable narratives from small rural communities, raw transcripts, audio recordings and full field notes are not publicly available. De-identified excerpts, the coding framework and methodological materials may be made available by the corresponding author upon reasonable request, subject to ethical and confidentiality restriction.

## Supplementary Materials

The following supporting information can be downloaded at: https://www.mdpi.com/article/10.3390/healthcare14121715/s1, Table S1: Thematic synthesis of barriers to dengue prevention and control.

## Abbreviations

The following abbreviations are used in this manuscript:

COMBI	Communication for Behavioural Impact
KAP	Knowledge, attitudes and practices
CIEIC-UPNW	Institutional Committee for Ethics and Scientific Integrity of Universidad Privada Norbert Wiener
