# Supplementary material for: Behavioral, Sociocultural, and Institutional Barriers to Dengue Prevention and Control Among Rural Communities in the Peruvian Amazon
Source: Healthcare (Basel). 2026 Jun 15;14(12):1715. doi: 10.3390/healthcare14121715 (PMC13300258; doi:10.3390/healthcare14121715)
Supplement: Supplementary file 1 [file healthcare-14-01715-s001.zip › healthcare-4348340-supplementary.pdf]

Supplementary Table S1. Thematic synthesis of barriers to dengue prevention and control

Domain	Category	Corpus descriptor
**Study setting**	Settlement A: roadside agricultural community with greater commercial movement	49 participants
	Settlement B: riverine community with irregular water supply and dispersed households	38 participants
	Settlement C: remote rural community with limited transport to the nearest health facility	33 participants
**Participant profile**	Household adult or primary caregiver	71 participants
	Community leader, neighborhood representative or local authority	15 participants
	Community health agent or health promoter	11 participants
	Health personnel or vector-control worker	10 participants
	Teacher, school representative or youth/community organizer	7 participants
	Municipal or other local institutional actor	6 participants
**Fieldwork material**	Individual in-depth interviews	84 records
	Focus group discussions	6 groups / 36 participants
	Household and community observation records	22 records
	Institutional communication materials reviewed	13 materials
	Local plans, meeting minutes or operational documents reviewed	7 documents

Note. This table describes the internal composition of the qualitative corpus and the fieldwork materials reviewed. Counts are included only to characterize the scope and diversity of the qualitative material. They should not be interpreted as prevalence, population frequency, statistical weight, representativeness, ranking or association. The analysis was based on triangulation across interviews, focus group discussions, observations and reviewed materials.
